# Common ground for biodiversity and ecosystem services: the “partial protection” challenge

**DOI:** 10.12688/f1000research.1-30.v1

**Published:** 2012-10-16

**Authors:** Daniel P Faith

**Affiliations:** 1 The Australian Museum, Sydney, Australia

## Abstract

New global initiatives require clarity about similarities and differences between biodiversity and ecosystem services. One argument is that ecosystem services capture utilitarian values, while biodiversity captures intrinsic values. However, the concept of biodiversity equally emerges from anthropogenic use values. Measures of biodiversity indicate broad option values, and so provide different information about future uses and benefits. Such differences nevertheless can be the basis for “common ground” for biodiversity and ecosystem services. Systematic conservation planning and related frameworks acknowledge such differences through effective trade-offs and synergies among different values of society. The early work on regional biodiversity trade-offs includes a little-explored aspect that could enhance this common ground. Regional planning here takes into account the “partial protection” of biodiversity provided by some land uses. Common-ground will be promoted by better integrating the ecosystem services and biodiversity conservation offered by ecosystems at the “natural end of the spectrum” with the partial protection and other benefits/services provided by more intensively-transformed places.

## Introduction

"Biodiversity" and "ecosystem services" increasingly travel together as companion terms. Examples include the new "Intergovernmental science-policy platform on biodiversity and ecosystem services", ( IPBES), the new Strategic Plan of the Convention on Biological Diversity ( CBD), and the emerging Global Biodiversity Observation Network ( GEO BON). These new initiatives require clarity about the similarities and differences between biodiversity and ecosystem services. Some distinctions naturally emerge from our basic definitions – "biodiversity" refers to living variation, and "ecosystem services" refers to benefits to humans from natural ecosystems. However, biodiversity also has traditional links to benefits/values, and here comparisons with ecosystem services continue to raise important issues.

Biodiversity sometimes is characterised as all about intrinsic, non-anthropogenic values, with ecosystem services then providing the links to human well-being. For example, Haines-Young and Potschin ^1^ argue: "Biodiversity has intrinsic value and should be conserved in its own right. However, the utilitarian arguments which can be made around the concept of ecosystem services and human well-being are likely to become an increasingly central focus of future debates about the need to preserve ‘natural capital’". Similarly, Hardy ^2^ argues: "The idea of ecosystem services allows for acknowledging more than the "intrinsic" value of biodiversity by expanding the breadth of the conservation argument to include the "utilitarian" values of nature". Thus, an argument is that only through ecosystem services do we move beyond biodiversity’s intrinsic values to also consider utilitarian values.

## Common ground

A recent statement by Reyers *et al.* ^3^ that "the concept of biodiversity emerges from an intrinsic context" echoes earlier studies, including the previous assertion by Reyers and colleagues ^4^ that "biodiversity and ecosystem services are associated with different values (intrinsic vs. utilitarian)" (see also ^5^). However, Reyers *et al.* ^3^ do suggest "common ground" based on biodiversity’s additional links to ethical, spiritual, and religious values. They argue that, because these are ecosystem services, conservation of ecosystems services sometimes captures biodiversity and its values (see also ^6, 7^).

In a response to Reyers *et al.*, Faith ^8^ points out that the concept of biodiversity equally emerges from anthropogenic use values, citing the early calls for conservation of diversity to ensure benefits "for present and future use" ^9^, and the early references ^10^ to "option values" (the value of biodiversity in providing uses, often unanticipated, for future generations; see also ^11, 12^). Thus, in contrast to recent perspectives, there is no requirement to add-in ecosystem services considerations in order to build a case for biodiversity conservation based on human-use values.

Reyers *et al.* ^13^ agree that the concept of biodiversity emerges from anthropogenic values. However, they object to Faith’s observation ^8^ that biodiversity and ecosystem services "may differ in how well they capture current and future uses". Reyers *et al.* correctly argue that ecosystem services include future uses. However, Faith argues that option values of biodiversity are broad in reflecting unknown benefits, including those from unknown elements or services ^14^. In contrast, ecosystem services typically focus on option values related to possible future use of known services (e.g. future timber from a forest area). For example, DIVERSITAS links option value to the "availability of a particular service for use in the future". Broader option values are measured by estimating biodiversity (for discussion see ^14, 15^). Thus, biodiversity by its nature arguably contributes something additional, something different, concerning potential future uses.

Reyers *et al.*'s ^13^ conclusion that "some scientists focus on differences while others focus on similarity and common ground" therefore is a concern. It implies that proposing differences is counter-productive to finding "common ground". However, I think any truly useful "common ground" for biodiversity and ecosystem services will build on differences. This is apparent in decision-support frameworks related to systematic conservation planning ^16^ and "regional sustainability analysis" ^17^ that seek trade-offs and synergies among the different values associated with biodiversity, ecosystem services, and other needs of society. Part of that common ground framework is now well-established. Measures of regional biodiversity are used to identify places with high versus low biodiversity marginal gains ("complementarity" values ^16^ which vary depending on other allocations in the region). For a given locality, high complementarity, combined with high co-benefits (or "negative costs" ^18, 19^) and low opportunity costs of conservation, implies priority for conservation over alternative land uses having higher costs and smaller co-benefits (for related work, see ^20– 27^ and Insights from an Australian planning framework for biodiversity and ecosystem services.

## Partial protection

The early foundations of that regional biodiversity-plus-costs framework ^17, 28– 30^ include some little-explored aspects that could enhance the common ground of biodiversity and ecosystem services: here, planning includes land/water uses offering ecosystem services or other benefits, combined with only "partial protection" of biodiversity (implying some lower complementarity value) ^17^. Early examples ^17, 19, 28^ illustrate cases where a partial protection option is allocated, and other cases where the non-conservation land use in a given place is preferred over the partial protection option because it maximises regional net benefits (see Partial degrees of protection and regional sustainability analysis).

The Millennium Ecosystem Assessment ^31^ (MA) highlights this approach in the context of biodiversity policy options:

"...an integrated biodiversity trade-offs framework (Faith *et al.* 2001a, 2001b) ^32, 33^ suggests how such partial protection (for example, from private land) can contribute to the region’s trade-offs and net benefits". However, the MA also observes that "The great uncertainty is about what components of biodiversity persist under different management regimes, limiting the current effectiveness of this approach" ^31^.

As more information of this kind becomes available, case studies should explore applications, and evaluate interesting variants of the partial protection framework. Variants now include extensions to the original DIVERSITY-ED ^17, 28– 30^, and TARGET (e.g., ^19^) partial protection approaches, to better accommodate multiple options for areas, and the related "partial protection" method in Marxan ^34^.

Because partial protection accommodates otherwise-competing values, it helps establish an inclusive, "common ground", framework that acknowledges differences. Biodiversity measures can complement ecosystem services in indicating broader option (and other) values. Further, "ecosystem services", which conventionally refer to ecosystems at the "natural end of the spectrum" ^35^, are complemented by more intensively-transformed places which sometimes provide partial protection along with other benefits/services.

Of course, one could define "ecosystem services" to capture all these aspects, but making clear distinctions helps to avoid possible conceptual confusions arising when everything is forced under the ecosystem services umbrella (where any human benefit from any place becomes an "ecosystem service"; for related discussion, see ^4, 7, 14, 36^). Ecosystem services can point to co-benefits specifically from retained natural ecosystems (providing essentially "full protection" of the elements of biodiversity in that place), and be integrated into a broader decision-support framework that also considers the partial protection (or no-protection) options in a region.
