# Supplementary material for: Common ground for biodiversity and ecosystem services: the “partial protection” challenge
Source: F1000Res. 2012 Oct 16;1:30. [Version 1] doi: 10.12688/f1000research.1-30.v1 (PMC3782344; doi:10.12688/f1000research.1-30.v1)
Supplement: Peer review history

# Peer review: Referee response for version 1

**Published:** 2012-10-22

**Authors:** Chris Margules

**Affiliations:** 1 CSIRO Ecosystem Sciences, Atherton, Australia

I am pleased to approve this article for publication. I have the following three brief comments:

**1.** It is important to acknowledge that partial protection, or protection of some components of biodiversity, is possible in places used predominantly for purposes other than biodiversity protection. The qualification of the MEA, noted by Faith, that it is difficult to measure such a contribution must also be acknowledged.

**2.** More intensively transformed places may also contribute ecosystem services even though they protect little or almost no biodiversity. For example, the sediment free water that sugar cane plantations can deliver (although that water may contain dissolved nutrients).

**3.** As a comment - an aside really - I wonder how anything can have non-anthropogenic values when those values are being assigned by people.

I have read this submission. I believe that I have an appropriate level of expertise to confirm that it is of an acceptable scientific standard.

# Peer review: Referee response for version 1

**Published:** 2012-10-22

**Authors:** Gary Luck

**Affiliations:** 1 Department of Wildlife Ecology and Management, Charles Sturt University, Albury, Australia

This brief but interesting opinion piece argues that biodiversity has both intrinsic value and human-use (utilitarian, instrumental) value.

Previous studies have focused primarily on the intrinsic value of biodiversity and assigned utilitarian values to the ecosystem services generated by biodiversity. Yet, Faith argues that biodiversity has utilitarian value beyond those of ecosystem services because protecting biodiversity yields option values for humans from unknown future benefits. That is, protecting biodiversity maximises future options for benefits that may be used in the future, but which we are unaware of now. Current benefits are captured in the ecosystem-services concept, and option value for ecosystem services focuses on future use of current known services. Hence, both biodiversity and ecosystem services provide utilitarian values, but in different ways.In essence, Faith argues for the need to recognise the differences between biodiversity and ecosystem services, and he promotes the use of the ‘partial protection’ approach within a systematic planning framework. The partial protection approach applied within a given region would consider the various trade-offs from protecting biodiversity and protecting land or systems generating services to maximise regional net benefits.

I agree with the need to recognise the different values and benefits of biodiversity and ecosystem services, and do not support subsuming biodiversity entirely within the ecosystem-services framework. The partial protection approach appears to offer promise in this area. This opinion piece is a reply to a reply and readers interested in following this argument should see citations 8 and 13 (in article reference list) and the original paper (Reyers, B *et al.* (2012) Finding common ground for biodiversity and ecosystem services).

I have read this submission. I believe that I have an appropriate level of expertise to confirm that it is of an acceptable scientific standard.
